# *pypet:* A Python Toolkit for Data Management of Parameter Explorations

**DOI:** 10.3389/fninf.2016.00038

**Published:** 2016-08-25

**Authors:** Robert Meyer, Klaus Obermayer

**Affiliations:** ^1^Neuroinformatics Group, Department of Software Engineering and Theoretical Computer Science, Technical University BerlinBerlin, Germany; ^2^Bernstein Center for Computational NeuroscienceBerlin, Germany

**Keywords:** parameter exploration, reproducibility, simulation, python, parallelization, grid computing

## Abstract

*pypet* (Python parameter exploration toolkit) is a new multi-platform Python toolkit for managing numerical simulations. Sampling the space of model parameters is a key aspect of simulations and numerical experiments. *pypet* is designed to allow easy and arbitrary sampling of trajectories through a parameter space beyond simple grid searches. *pypet* collects and stores both simulation parameters and results in a single HDF5 file. This collective storage allows fast and convenient loading of data for further analyses. *pypet* provides various additional features such as multiprocessing and parallelization of simulations, dynamic loading of data, integration of git version control, and supervision of experiments via the electronic lab notebook Sumatra. *pypet* supports a rich set of data formats, including native Python types, Numpy and Scipy data, Pandas DataFrames, and BRIAN(2) quantities. Besides these formats, users can easily extend the toolkit to allow customized data types. *pypet* is a flexible tool suited for both short Python scripts and large scale projects. *pypet*'s various features, especially the tight link between parameters and results, promote reproducible research in computational neuroscience and simulation-based disciplines.

## 1. Introduction

Numerical simulations are becoming an important part of scientific research. In computational neuroscience researchers create increasingly detailed models of neural phenomena. For instance, Reimann et al. (2013) simulated local field potentials in a neural network of more than 12,000 multi-compartmental cells. Similarly, Potjans and Diesmann (2014) built a full-scale spiking neuron network model of a cortical microcircuit. Such complex computational models pose a challenge to reproducibility in research. Many researchers rely on custom software and data formats. In addition, scripts and results of simulations are rarely shared, if ever. These conditions make numerical experiments hard to reproduce. Stodden (2011) even speaks of a “credibility crisis” of computational results. There is an ongoing debate about the mandatory publication of source code in scientific research (Ince et al., 2012).

Still, even the open availability of software does not guarantee reproducibility. Researchers are unlikely to use undocumented and unmaintained software solutions created by others. Even when researchers are willing to inspect source code, ill-documentation often prohibits them from successfully reimplementing published models (Topalidou et al., 2015). Furthermore, simulations are usually highly parameterized, with up to hundreds of parameters (Reimann et al., 2013; Potjans and Diesmann, 2014). Reproducing simulation results becomes challenging when the values of those parameters are not provided. This is not only a problem for published experiments but also for a scientist's own previous work. Researchers may fail to reproduce their own results due to missing parameters.

*pypet* is designed to address these problems in the management of numerical experiments. The two main goals of the software package are, first, to allow easy and flexible exploration of parameter spaces and, second, to jointly store parameters and results for each experiment.

*pypet* stands for **Py**thon **P**arameter **E**xploration **T**oolkit. It targets researchers and engineers executing numerical experiments of any kind; not only related to Neuroscience. Besides simulations of neural networks, other areas of applications could be parameter explorations for machine learning pipelines or simulations of complex systems like computational fluid dynamics. *pypet* supports simulations written in Python, a platform-independent programming language. Python is increasingly used in neuroscience (Muller et al., 2015) and other scientific disciplines (Fangohr, 2004; Bäcker, 2007; Borcherds, 2007; Lin, 2012; Meyerovich and Rabkin, 2013).

With *pypet* the user can explore arbitrary parameter spaces by simply specifying Python lists of parameter points. These points define individual simulation runs and lead to numerical results. Tight linkage of parameters and results is achieved by storing all data together in the convenient HDF5 format^1^. Besides, *pypet* provides various other features. Among these are native support for parallelization, methods to annotate data, and integration with git version control. A summary of *pypet*'s features is given in Box 1.

Box 1 **Main features**

- Novel tree container Trajectory, for handling and managing of parameters and results of numerical simulations
- Grouping of parameters and results into meaningful categories
- Accessing data via natural naming, e.g., traj.parameters.neuron.gL
- Automatic storage of simulation data into HDF5^2^ files via PyTables^3^
- Support for many different data formats
  - Python native data types: bool, int, long, float, str, complex
  - Python containers: list, tuple, dict
  - NumPy arrays and matrices (van der Walt et al., 2011)
  - SciPy sparse matrices (Oliphant, 2007)
  - Pandas Series, DataFrame, and Panel (McKinney, 2011)
  - BRIAN and BRIAN2 quantities and monitors (Goodman and Brette, 2008; Stimberg et al., 2014)
- Easily extendable to other data formats
- Exploration of the parameter space of one's simulations
- Merging of trajectories residing in the same space
- Support for multiprocessing, *pypet* can run simulations in parallel
- Analyzing data on-the-fly during multiprocessing
- Adaptively exploring the parameter space combining *pypet* with optimization tools like the evolutionary algorithms framework DEAP (Fortin et al., 2012)
- Dynamic loading of parts of data one currently needs
- Resuming a crashed or halted simulation
- Annotation of parameters, results, and groups
- Git integration, *pypet* can make automatic commits of one's codebase
- Sumatra integration, *pypet* can automatically add one's simulations to the electronic lab notebook tool Sumatra (Davison, 2012)
- *pypet* can be used on computing clusters or multiple servers at once if it is combined with the SCOOP framework (Hold-Geoffroy et al., 2014)

### 1.1. Existing software

In recent years a couple of software projects dealing with data management have been developed—especially targeted to researchers in computational neuroscience.

NeuroManager (Stockton and Santamaria, 2015) facilitates automated scheduling of simulations in MATLAB with heterogeneous computational resources. Such computational resources can range from simply using the host computer—from which scheduling was started—to a network of other computers or even clusters and computer grids. NeuroManager, written in object-oriented MATLAB, allows the user to specify simulations in terms of pure MATLAB code or MATLAB code wrapping existing simulators like NEURON (Carnevale and Hines, 2006). The parameter space defined by the simulators can be explored using NeuroManager's scheduling routine by utilizing heterogenous computing resources; granted these resources support the needed software requirements like MATLAB licenses. In contrast, *pypet* is written in Python and all of *pypet*'s requirements are open source and freely available.

Mozaik (Antolík and Davison, 2013) is a Python data management toolkit especially designed for network simulations of two-dimensional neural sheets. It relies on the simulator environment PyNN (Davison, 2008). Its design goals are similar to *pypet*'s. Mozaik aims for integrating parameters and model descriptions with the simulator execution as well as the storage of results. However, the focus on two-dimensional networks makes it less flexible in comparison to *pypet*.

Lancet (Stevens et al., 2013) constitutes a more general approach to workflow management and integrates with IPython notebooks (Perez and Granger, 2007). Lancet is a well-designed alternative to *pypet*, especially for smaller projects that fit into the scope of a single notebook. Like *pypet*, Lancet is simulator agnostic. It even allows to interact with other programs not written in Python as long as these can be launched as processes and return output in form of files. Hence, Lancet does not store the user data directly but assumes that results are written into files. Accordingly, given large parameter explorations with many simulation runs, the user may end up with her data scattered among many different files. This can be cumbersome to manage and may complicate the analysis of results. In contrast, *pypet* directly stores parameters and results side by side into a single HDF5 file.

VisTrails (Bavoil et al., 2005) is a workflow and provenance management system written in Python that focuses on automation of visualizations. It is mainly operated through a graphical user interface (GUI) and targets an audience less akin to programming. *pypet* offers no GUI, but it is a Python library that users can import and use in their own source code to write scripts and programs. Hence, *pypet* is more flexible in comparison to VisTrails. It is suitable for researchers that need low-level management of project code and their numerical data, of course, at the cost of requiring programming experience in Python.

The primary goal of Sumatra (Davison, 2012) is to enhance reproducible research. Sumatra serves as an electronic lab notebook. The command line program does not only link all simulation parameters to result files, but also keeps track of the entire computing platform. It stores information like the used operating system and particular versions of software dependencies. Sumatra can be nicely integrated with *pypet* to automatically trigger a Sumatra record with every simulation start. The combination of Sumatra and *pypet* allows for comprehensive provenance management. Accordingly, users can track soft- and hardware dependencies with Sumatra as well as store simulation parameters and results tightly linked in a single file via *pypet*.

## 2. *pypet* architecture and development

In the following we will discuss general design principles of *pypet* and layout the architecture and structure of the Python package. First, we are going to start with *pypet*'s packaging and adhesion to the concept of test driven development. Next, we will present our conceptualization of parameter explorations. Furthermore, we are going to introduce the general layout followed by more detailed descriptions of the individual components. Lastly, we will finish with some use case examples.

### 2.1. Packaging and testing

*pypet* is a pure Python *package*^4^ and supports Python versions 2.6, 2.7, 3.3, 3.4, and 3.5. It is platform independent and runs under Linux, Windows, and OS X with 32-bit as well as 64-bit architectures. The package is modularized and *pypet* is designed following the concept of object oriented programming^5^.

Furthermore, the source code is openly available and hosted on the prominent *github*^6^ code sharing platform. In addition, *pypet* is bundled on the Python Package index^7^ (PyPI) to allow fast and easy installation using the package managing system *pip*.

Besides comprehensive documentation, it is important for software packages—scientific ones in particular—that all functionality is well tested (Gewaltig and Cannon, 2014). Therefore, *pypet* is designed using test driven development. Accordingly, small features and single functions are already accompanied with corresponding test cases. In addition, we apply continuous integration testing. Every addition of new code triggers a full battery of package wide tests which are automatically started and deployed on independent build servers. *pypet* is tested using the services Travis-CI^8^ with a Linux environment and AppVeyor^9^ providing Windows servers. Every time a new code addition is pushed to the code repository on github, the unit and integration tests are automatically deployed by Travis-CI and AppVeyor. This guarantees that new features do not break existing functionality. In addition to continuous integration testing, we use the coveralls^10^ web service to quantify how comprehensive the test suite is. As of July 2016, *pypet*'s core modules encompass about 10,000 lines of pure Python code of which more than 90% are hit by the test battery that already exceeds 1000 tests.

Besides the comprehensive test battery, *pypet* has been successfully used in our research group for parameter explorations of large neural networks. The toolkit helped managing several ten thousand simulation runs and HDF5 files with sizes of more than hundred gigabytes.

### 2.2. Parameter exploration and conceptualization

*pypet*'s goals are to provide side by side storage of results as well as parameters and to allow for easy parameter exploration. Our definition of a parameter exploration is as follows: It is the process of sampling an *n*-dimensional parameter space of a simulation or model implementation with a pre-defined set of points within the space. Running the simulation or model independently with each point in the parameter space produces further data. This data is considered to be results. The dimensions of the parameter space can be heterogeneous, i.e., these may encompass integers, real values, or even less mathematical concepts like Python tuples, which are immutable list like data structures. Therefore, we also refer to a dimension of the parameter space simply as a “parameter.”

Moreover, we assume that from the *n*-dimensional space usually only a much smaller sub-space is sampled of size *n*′ with *n*′ ≪ *n*. Accordingly, most parameters are fixed and only a minority are varied and explored. For instance, the visual cortex network model by Stimberg et al. (2009) is based on several tens of parameters, but the authors varied only two of these comprehensively.

Furthermore, the set of points is sequentially ordered. The order may be arbitrary, but it is fixed such that the *i*th point in the parameter space corresponds to the *i*th run of a simulation or model. Due to the order, one may not just think of sampling the parameter space, but rather following a discrete trajectory through the space. Accordingly, the top-level container managing all parameters and results is called Trajectory.

**Figure 1:**
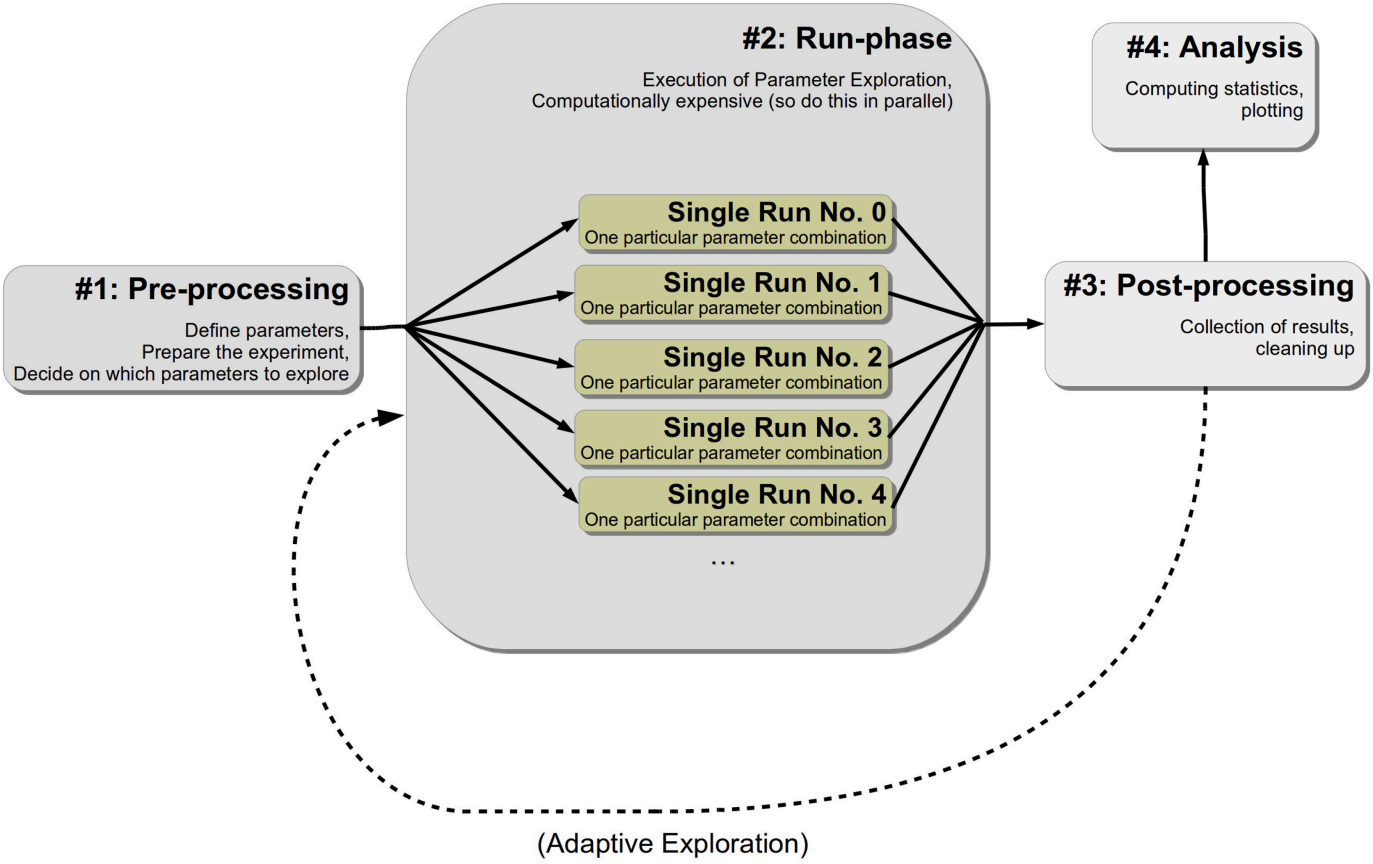
**Conceptualization of a simulation or numerical experiment**.

Next, we will briefly discuss a particular layout of simulations that fits best with *pypet*. This conceptualization is also sketched in Figure 1. We assume that numerical experiments or simulations usually comprise between two to four different stages or phases:

1. **Pre-processing**: Parameter definition, preparation of the experiment
2. **Run phase**: Fan-out structure, usually parallel running of different parameter settings, gathering of individual results for each single run
3. **Post-processing** (optional): Cleaning up of the experiment, sorting results, etc.
4. **Analysis of results** (optional): Plotting, calculating statistics, etc.

The first pre-processing stage can be further divided into two sub-stages. In the beginning the definition of parameters is given and, secondly, one's experiment is initialized and configured. Conceptually, the addition of parameters can be implemented by a distinct function or even by another script for re-usability. Moreover, the first phase also involves the decision on how the parameter space is explored. Configuration and initialization might encompass creating particular Python objects or pre-computing some expensive functions that otherwise would be computed redundantly in every run in the next phase.

The second stage, the run phase, is the actual execution of one's numerical simulation. All different points in the parameter space that have been specified before for exploration are tested on the model. As a consequence, one obtains corresponding results for all parameter combinations. Since this stage is most likely the computationally expensive one, one probably wants to parallelize the simulations. We refer to an individual simulation execution with one particular parameter combination as a **single run**. Because such single runs are different individual simulation executions with different parameter settings, they are completely independent of each other. The results and outcomes of one single run should not influence another. This does not mean that non-independent runs cannot be handled by *pypet*; they can. However, keeping single runs independent greatly facilitates the parallelization of their execution.

Thirdly, after all individual single runs are completed one might perform post-processing. This could involve merging or collection of results of individual single runs or deleting some sensitive Python objects. In case one desires an adaptive or iterative exploration of the parameter space, one could restart the second phase. In this case the Trajectory can be extended. The user can iteratively add some more points of the parameter space and alternate the run phase and post-processing before terminating the experiment. The iterative approach may be based on some optimization heuristics like DEAP evolutionary algorithms (Fortin et al., 2012). *pypet*'s online documentation provides a comprehensive example on how to use both libraries together. Note *pypet* is useful for optimization tasks where the resulting trajectory through the parameter space or intermediate results should be stored for later analysis. If the user does not care about these, but she is only interested in the final best parameters, DEAP—alone or in combination with BluePyOpt (Van Geit et al., 2016) for optimizing neural models—is already sufficient.

Fourthly, one may desire to do further analysis of the raw results obtained in the previous phases. This constitutes the final stage of an experiment and may include the generation of plots or calculation of statistics. For a strict separation of experimental raw data from its statistical analysis, one is advised to separate this final phase from the previous three. Thus, this separation could mean starting a completely different Python script than for the phases before.

### 2.3. General package structure

**Figure 2:**
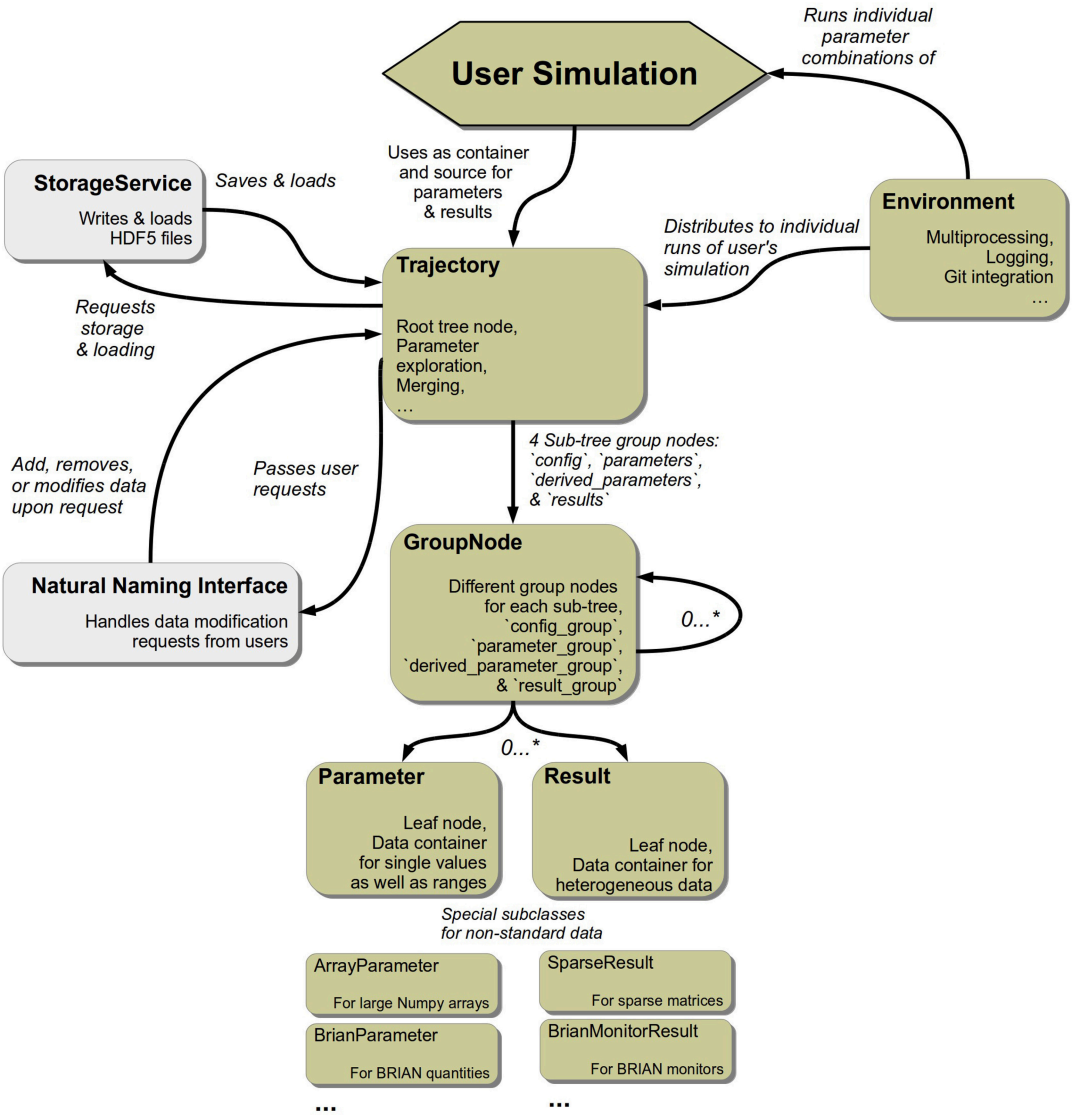
**Main components of ***pypet*****. Elements in light gray mark objects that operate in the background with no direct user interaction.

*pypet* encompasses five key modules. The trajectory.py module contains the Trajectory class that constitutes the main data container the user interacts with. User requests to a Trajectory are passed onto and processed by a service called NaturalNamingInterface residing in the naturalnaming.py module. Moreover, the Trajectory allows the arbitrary exploration of the parameter space and manages all data including parameters, results, as well as configuration specifications. All of these are further encapsulated by their own abstract containers which can be found in the parameter.py module. In case data is stored to disk, this is handled by the HDF5StorageService located in the storageserivce.py module. Currently, the data is saved in the HDF5 format. Storage and loading of trajectories follow well-defined application programming interfaces (API). Hence, the implementation of other backends, like SQL or MongoDB^11^ for example, is possible without the need to change any other *pypet* core code. Finally, the environment.py module provides the so called Environment object for handling the running of simulations. This general structure of the *pypet* components is sketched in Figure 2.

### 2.4. Parameters and results

The parameter.py module provides the so called Parameter class that follows a base API called BaseParameter. The Parameter contains data that is explicitly required as parameters for one's simulations. For the rest of this manuscript we follow the convention that the stylized Parameter denotes the abstract container. The not stylized expression “parameter” refers to the user data that is managed by the Parameter container. This notation holds analogously for user results encapsulated by the Result class. This class implements the base API BaseResult.

Parameters follow two main principles. First, a key concept of numerical experiments is the **exploration** of the parameter space. Therefore, the Parameter containers do not only manage a single value or data item, but they may also take a range of data items. Elements of such a range reside in the same dimension, i.e., only integers, only strings, only NumPy arrays, etc. The exploration is initiated via the Trajectory. This functionality will be introduced shortly. Secondly, a Parameter can be **locked**; meaning as soon as the Parameter container is assigned to hold a specific value or data item and the value or data item has already been used somewhere, it cannot be changed any longer and becomes immutable (except after being explicitly unlocked). This prevents the cumbersome error of having a particular parameter value at the beginning of a simulation, but changing it during runtime by accident. Such an error can be difficult to track down.

Parameter containers accept a variety of different data items, these are

- Python natives (int,str,bool,float,complex),
- NumPy natives, arrays and matrices of type np.int8 to np.int64, np.uint8 to np.uint64, np.float32, np.float64, np.complex, and np.str
- Python homogeneous non-nested tuples and lists

For more complex data, there are specialized versions of the Parameter container. For instance, the SparseParameter is a container for SciPy sparse matrices (Oliphant, 2007) and the BrianParameter can manage quantities of the BRIAN simulator package (Goodman and Brette, 2008).

Moreover, Result containers are less restrictive than Parameters in terms of data they accept. They can also handle Python dictionaries, the Python implementation of a hash map, and Pandas DataFrames (McKinney, 2011), a tabular data structure.

Similar to the Parameter, there exist specialized versions of a Result, like a SparseResult. In case the user relies on some custom data that is not supported by the Result, Parameter, or their specialized descendants containers, the user can implement a custom solution. Customized containers are straightforward and only need to follow the API specifications given by BaseResult and BaseParameter.

### 2.5. Trajectory

The Trajectory is the container for all results and parameters of one's numerical experiments. The Trajectory instantiates a tree with **groups** and **leaf nodes**. The instantiated Trajectory object itself is the root node of the tree. The leaf nodes encapsulate the user data and are the Parameter and Result containers. Group nodes cannot contain user data directly, but may contain other groups and leaf nodes. By using only groups and leaves there cannot be any cycles within the trajectory tree. However, one can introduce **links** that refer to other existing group or leaf nodes.

Results can be added to the Trajectory tree at any time. Parameters can only be introduced before the individual simulation runs are started. Both, parameters and results, can be recovered from the trajectory tree at any time, for example if needed during a simulation run or later on for data analyses. The user data can be recalled using natural naming, i.e., the user can rely on the Python dot notation familiar from object oriented programming. Such natural naming requests are handled by the NaturalNamingInterface class in the background.

Exploration of the parameter space is initiated using the Trajectory as well. The user simply passes a Python dictionary containing the parameter names as keys and lists of the corresponding data ranges they like to explore as values. For a thorough grid-like exploration there exists the functionality to create the Cartesian product set of multiple parameters.

### 2.6. Data storage and loading

Storage and loading of the Trajectory container and all its content are not carried out by the Trajectory itself but by a service in the background. Currently, all data is stored into a single HDF5 file via the HDF5StorageService. To interface HDF5, the storage services uses the PyTables library^12^.

The acronym HDF5 stands for the fifth version of the *Hierarchical Data Format*. It is a convenient format because it allows compressed reading and writing of data to the hard disk with high performance. More important, as its name suggests, data is ordered in hierarchies that are similar to the file and folder structure of most operating systems. The data hierarchies and the numerical data therein can be directly inspected with tools like *HDFView*^13^. Not surprisingly, the tree structure of the Trajectory is mapped one-to-one to the hierarchical structure in the HDF5 file.

Usually, the storage of a Trajectory is automatically triggered by *pypet* in regular intervals. Additionally, the user can manually initiate storing and loading if desired. Moreover, *pypet* supports automatic loading of data as soon as the user needs it. No explicit loading is necessary and data is recovered from the HDF5 file on-the-fly.

### 2.7. Environment

The Environment defines a scheduler for the numerical experiments. It constitutes a general framework in which the user can embed her simulations. It allows the user to disentangle the core simulation from administrative tasks like distribution and repeated execution of runs and data serialization.

The Environment can be used to trigger independent simulation runs according to the exploration specified in the Trajectory container. *pypet*'s combination of the Trajectory and the Environment to start the simulation runs is more convenient and flexible than brute-force approaches such as bash scripts passing parameters as command line arguments or nested for-loops in Python scripts. Accordingly, *pypet* allows to easily change between different parameters or sets of parameters for exploration without rewriting large segments of the code or the need for new bash scripts. Besides more flexible exploration, *pypet* offers other convenient features. For example, the Environment natively supports multiprocessing and parallelization of simulation runs.

Moreover, in case of long running simulations or many runs, the Environment notifies the user about the progress and gives an estimate of the remaining time in regular intervals. Furthermore, the Environment will automatically trigger the storage of results after every individual simulation run. In addition, it monitors the simulation execution in terms of keeping log-files. By default, general log-files are accompanied by specialized error logs. The latter display only error messages to allow easier identification and debugging in case there are errors in the user's simulation.

## 3. Usage

So far we have introduced *pypet*'s main components and sketched their functionality. In this section we will provide information about the installation and some usage examples.

The usage examples are based on *pypet* version 0.3.0. Although we aim for a stable API, the reader is always advised to check the current online documentation^14^.

### 3.1. Installation

Because *pypet* is a pure Python package, its installation is straightforward and does not require more involved steps like compilation of source code. If the Python package manager *pip* is available^15^, one can simply install *pypet* from the command line:


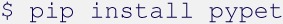


Alternatively, one can download *pypet* from the PyPI^16^ web page, unpack it, and run


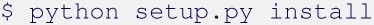


in a terminal.

Note that *pypet*'s four core prerequisites are NumPy, SciPy, PyTables, and Pandas. These are standard libraries in scientific Python and have most likely been installed already on many computer systems. For a fresh Python environment, however, one needs to install these before setting up *pypet*.

### 3.2. Naming convention

We implemented a general naming convention that applies to the Trajectory, all groups, and all containers that can encapsulate user data, i.e., the Result and Parameter introduced before. To avoid confusion with the natural naming scheme and the functionality provided by the Trajectory, we use prefixes. This idea is taken from the software package PyTables. We use f_ for methods and functions and v_ for Python variables, attributes, and properties.

For instance, given a particular instantiated Result denoted by the variable myresult, myresult.v_comment refers to the object's comment attribute and myresult.f_set(mydata=42) is the function for adding data to the Result container. Whereas, myresult.mydata can be a data item named mydata provided by the user.

### 3.3. Basic example

Here we are going to describe a basic usage example. We will simulate the multiplication of two values, i.e., *z* = *x* · *y*. Before discussing the details of the simulation, we provide the full script below for an overview:


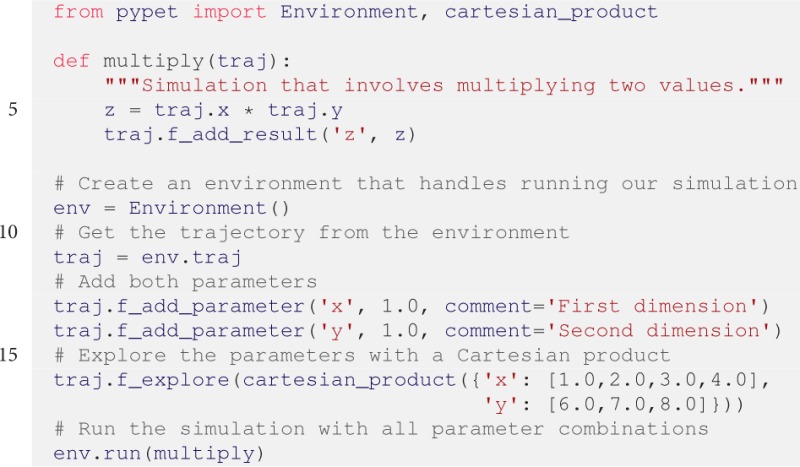


First, we consider the top-level simulation function that contains the user's core simulation code. The function needs to take the Trajectory container as the first argument. It is allowed to take other positional as well as keyword arguments if needed.

In this example the top-level simulation function multiply is defined as


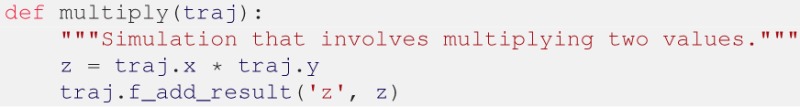


The function makes use of a Trajectory container traj which manages our parameters. Because multiply is evoked with all points in the parameter space, here traj holds a particular choice of *x* and *y*. We can access the parameters simply by natural naming, i.e., using Python's dot notation, as seen above via traj.x and traj.y. Note that the full paths of the parameters in the trajectory tree are traj.parameters.x and traj.parameters.y, respectively. However, to spare the user an excessive amount of typing, the Trajectory supports so called *shortcuts*. If the user leaves out intermediate groups in the natural naming request (here the group parameters), a fast search is applied by the NaturalNamingInterface to find the requested items down the tree (here the leaves x and y).

Subsequently to computing z = traj.x * traj.y, the value of z is added as a result to the traj container via traj.f_add_result('z', z). This concludes the simple top-level simulation function.

After the definition of the job that we want to simulate, we create an Environment denoted by env that runs the simulation. Hence, we start with the first phase of the simulation conceptualization, the initialization of the experiment and addition of parameters. Moreover, we do not pass any arguments to the constructor and simply use *pypet*'s default settings for an Environment:


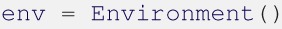


The Environment will automatically generate a Trajectory which we can access via the env.traj property. Next, we can populate the container with the parameters. We add them using default values *x* = *y* = 1.0:


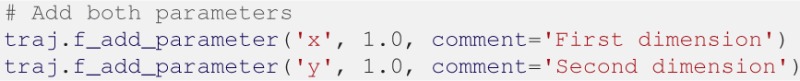


Additionally, one can provide a descriptive comment to inform potential other users or researchers about the parameter's scope and meaning.

Note for simplicity here parameter addition is done in the main script. In order to re-use parameter definitions it can be useful to outsource this addition into a distinct Python function that can be imported upon need.

Afterwards, we decide upon how to explore the parameter space. More precisely, we are interested in the Cartesian product set {1.0, 2.0, 3.0, 4.0} × {6.0, 7.0, 8.0}. Therefore, we use f_explore() in combination with the builder function cartesian_product(). The f_explore() function takes a dictionary with parameter names as keys and lists specifying the parameter exploration ranges as values. Note that all lists need to be of the same length unless using cartesian_product(). In this case the list lengths may differ because the cartesian_product() function will return the Cartesian product yielding lists with appropriately matching lengths:


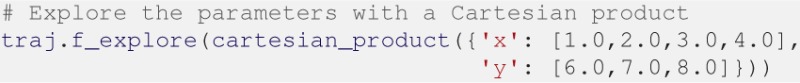


Finally, we need to tell the Environment to run our job multiply with all parameter combinations:


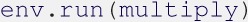


This will evoke our simulation twelve times with the parameter points (1.0, 6.0), (2.0, 6.0), …, (4.0, 8.0). This processing of all parameter combinations corresponds to the fan-out structure of the second phase. The Trajectory and all results are automatically stored into an HDF5 file. By default *pypet* sorts all results automatically in the sub-trees results.runs.run_XXXXXXXX, where XXXXXXXX is the index of the run; run_00000002 for the second run, for example. This tree structure is not mandatory, but can be changed and modified by the user. For details the reader is directed to the online documentation.

Note this storage scheme scatters data across the HDF5 file. For such a simple scenario where the result is only a single floating point number this produces some overhead. If desired, this overhead can be avoided by collecting all results before storing, see also Section 4.1.

In this basic example this could be implement as follows. The function multiply could simply return the value z:


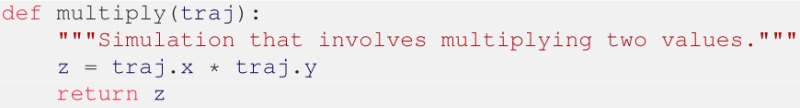


In this case all the results are collected by the environment. Accordingly, the Environment's run() function returns a sorted list of tuples where the first entry is the index of the run followed by the returned data: [(0, 6.0), (1, 12.0), …, (11, 32.0)]. Note that *pypet* starts counting run indices at 0. All data could be stored as a list using manual storing in a short post-processing step:


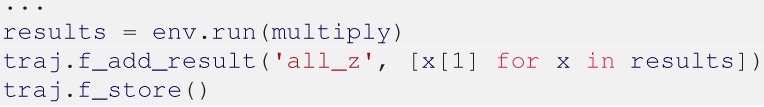


### 3.4. Cellular automata simulation

We will demonstrate how to use *pypet* in the context of a more sophisticated simulation. We will simulate one-dimensional elementary cellular automata (Wolfram, 2002). Celullar automata are abstract computational systems that can produce complex behavior based on simple transition rules. An automaton consists of a finite sequence of *n* ordered cells: $s_{0}^{t}s_{1}^{t}\ldots s_{n-1}^{t}$. Each cell can take two possible states 0 and 1, i.e., $s_{i}^{t}\in \left\{0,1\right\}$. The states are updated in *k* discrete time steps, i.e., *t* = 0, 1, …., *k* − 1, according to some transition rule function *f*. The updates depend only on the immediate neighborhood of a cell, that is the cell's current state and the states of its direct left and right neighbors:

(1) $$s_{i}^{t+1}=f(s_{i-1}^{t},s_{i}^{t},s_{i+1}^{t}).$$

Hence, there exist 256 different transition rules. Boundary conditions are periodic, i.e., $s_{0}^{t+1}=f\left(s_{n-1}^{t},s_{0}^{t},s_{1}^{t}\right)$ and $s_{n-1}^{t+1}=f\left(s_{n-2}^{t},s_{n-1}^{t},s_{0}^{t}\right)$. For example, the prominent rule 110, that is proven to be Turing complete (Cook, 2004), follows the state updates specified in Table 1. The name 110 stems from the decimal conversion of the update steps ordered according to the binary states of the neighborhood.

**Table 1:** **Transition function ***f*** of rule 110**.

Current state $s_{i-1}^{t}s_{i}^{t}s_{i+1}^{t}$	111	110	101	100	011	010	001	000
Next state $s_{i}^{t+1}$	0	1	1	0	1	1	1	0

*The rule is named after the decimal conversion of the binary representation: 110 = 2^6^ + 2^5^ + 2^3^ + 2^2^ + 2*.

The Python implementation of a cellular automaton with random initial conditions is given below:


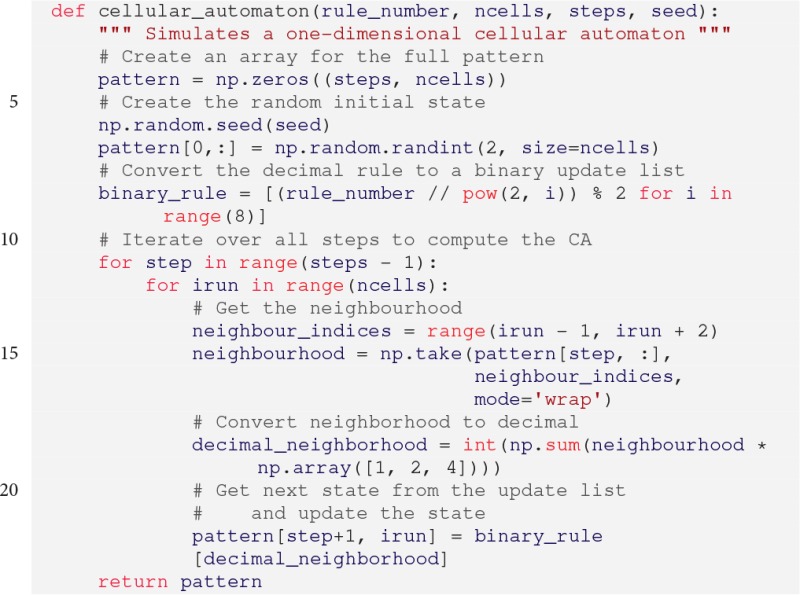


The parameters are the rule number (rule_number, decimal representation of *f*), the number of cells (ncells, *n*), the number of time steps (steps, *k*), and the seed for the random number generator (seed). The details of the implementation do not matter here. The important part is that the function cellular_automaton () returns the full cell pattern containing the states of all cells at all time steps (line 22).

Given such existing simulator functionality that takes parameters and returns a result, *pypet* can be added to operate on top of the code base. One simply needs a wrapper function that passes parameters from and results back to *pypet*:


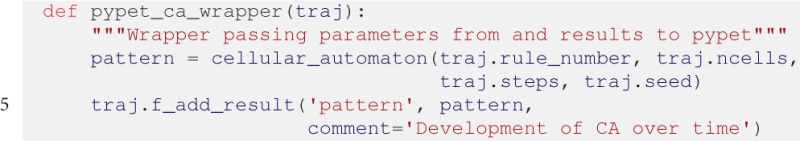


Still, some boiler-plate code is missing to add parameters, decide what to explore (here different transitions rules), and start the simulation:


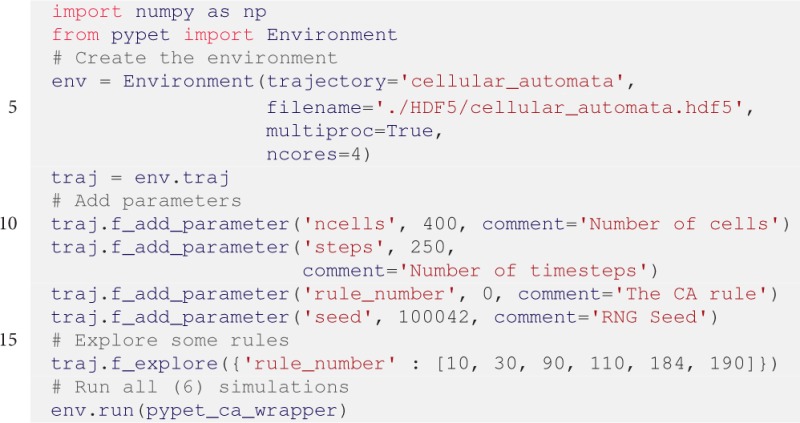


In contrast to the previous example, we passed some keyword arguments to the Environment constructor. We use trajectory = 'cellular_automata' and filename = './HDF5/cellular_automata.hdf5' to explicitly specify the Trajectory's name and the resulting HDF5 file. Moreover, *pypet* natively supports parallelization using the Python multiprocessing library. As shown above, to run all simulation runs on four cores at the same time, we pass the multiproc=True and ncores=4 keywords.

Next, we want to plot the results. According to the conceptualization introduced previously, we assume that this phase is performed in a different script and it is independently executed from the previous simulation. The full script reads:


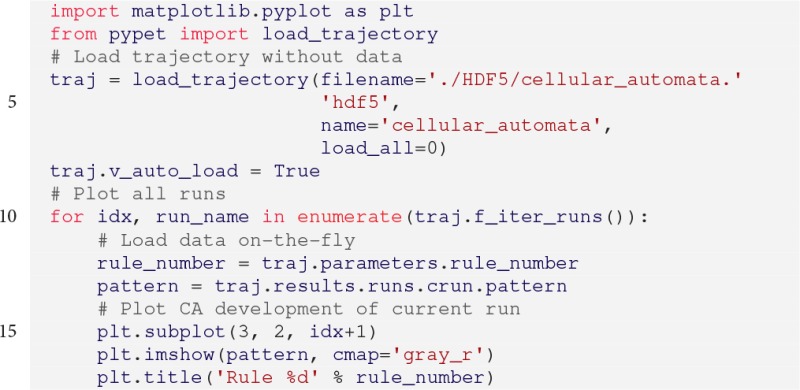


**Figure 3:**
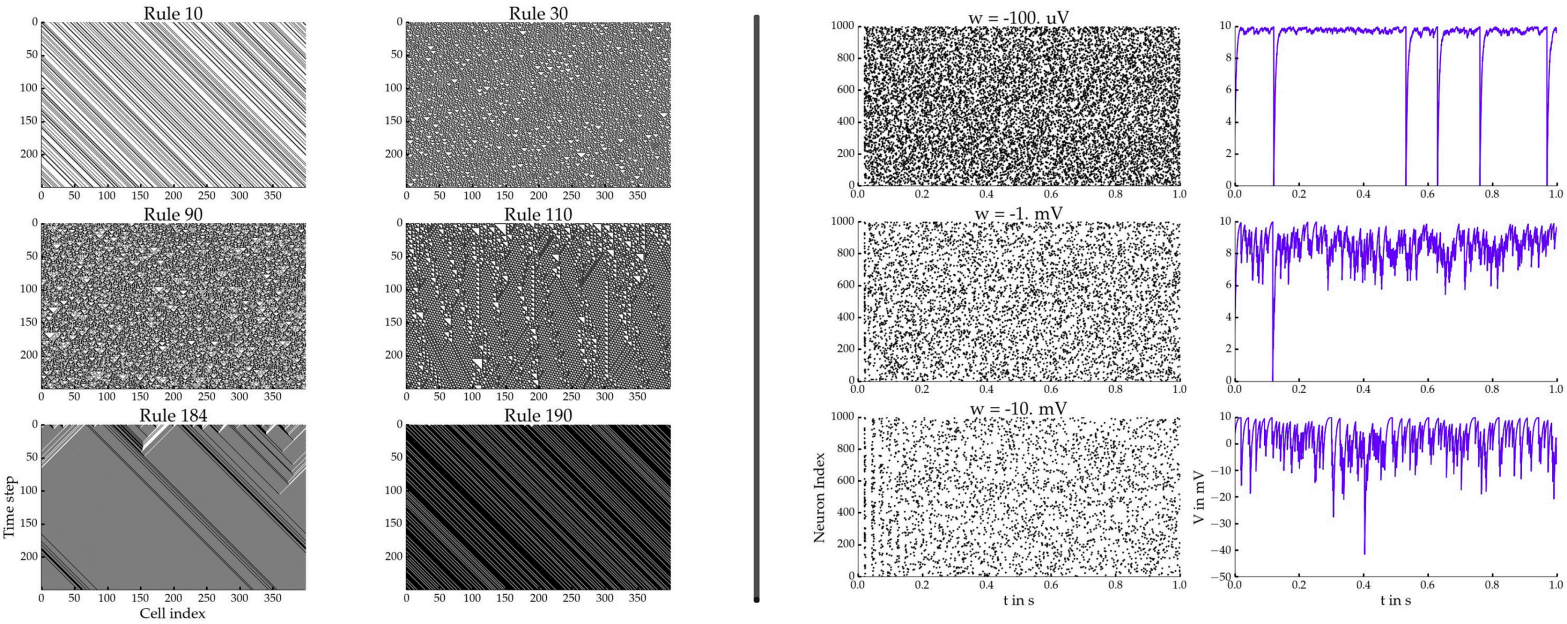
**Left:** Cellular automata simulation showing six different rules. **Right:** BRIAN2 network simulations for three different synaptic weights *w*. Spiking raster plots on the left and the voltage traces of the 0th neuron on the right.

The corresponding plots are shown on the left hand side of Figure 3.

We use the load_trajectory() function to recover the container from the HDF5 file. Note the keyword load_all=0 which enforces *pypet* to only load the root node of the tree and skip the rest of the data. This is particularly useful if our data is large, potentially hundreds of gigabytes. Thus, we do not load all data on start-up, but only when we need it; hence the statement traj.v_auto_load = True in line 7. This allows loading of data on-the-fly without explicit user request.

Moreover, the method traj.f_iter_runs() (line 9) iterates all runs (here 6) sequentially and modifies all explored parameters accordingly (here only rule_number). Hence, the explored parameter rule_number is iteratively set to its explored value of the corresponding run. This is helpful for natural naming requests which will return the value of the current run iteration. Consequently, traj.parameters.rule_number (line 11) will return 10 in the first loop iteration, followed by 30, 90, 110, 184, and 190.

This applies analogously to the statement traj.results.runs.crun.pattern (line 12) to return the cell pattern of each run. Due to traj.v_auto_load = True (line 7), there is no explicit loading with the Trajectory's f_load() function necessary, but *pypet* loads the cell patterns in the background as soon as the natural naming request traj.results.runs.crun.pattern is processed. If because of such a naming request a new node or new data is required from the trajectory or one of its nodes that is not part of the current tree in memory, *pypet* will hand over the request to the storage service. The service loads data from the HDF5 file and adds it to the tree. In addition, one may notice the identifier crun, short for *current run*. As mentioned before, by default, all results added via f_add_result() during a single run are automatically sorted into the Trajectory tree in the branch *results.runs.run_X*, where *X* is the index of the corresponding run. In combination with f_iter_runs(), crun maps always to the run currently processed within the for-loop. In the first iteration this is the 0th run, *run_0*, followed by *run_1* and so on.

As a side remark, instead of using f_iter_runs(), one can manually set a Trajectory to a particular run via traj.v_idx = 2, for example. As a consequence, all explored parameters are set to the values of the second run and crun maps to *run_2*. For undoing this and to recover the default settings, one writes traj.v_idx = -1. Indeed, this internal pointer v_idx is also used by f_iter_runs() and iteratively set to each run.

Moreover, the user does not have to iterate through all runs to find particular parameter combinations. *pypet* supports searching the trajectory for a particular run or subsets of runs via lambda predicate filtering. For example,


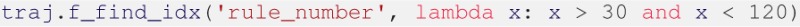


searches for run indices where the decimal rule representation is larger than 30 but smaller than 120 (here runs 2 and 3).

### 3.5. Post processing and adaptive exploration

Here we will demonstrate how one can alternate the second and third stage, the run and post-processing phases, respectively, to adaptively explore the parameter space. We will use a simple stochastic optimization to maximize the function

(2) $$f(x)=-{\left(x+4\right)}^{6}+5{\left(x-10\right)}^{4}-2{\left(x-4\right)}^{2}+x.$$

We will iterate generations of parameter points. The points will be randomly sampled from normal distributions with their centers located at the best points of previous generations. Thereby, we optimize the function in a greedy manner by exploring the local neighborhood of the current best point. Note that there are much more efficient ways for stochastic optimization than demonstrated here, but this should only serve as an example.

Our top-level simulation function reads:


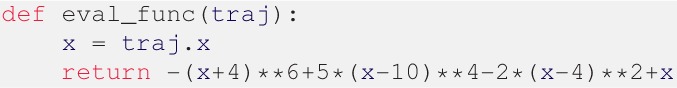


We do not want to store every computed value, but simply pass the results to the outer scope for post-processing. Accordingly, instead of using the f_add_result() functionality of the Trajectory, the simulation eval_func returns the result.

Next, we need to create an Environment, add the parameters, add the parameter exploration, and alternate the simulation runs with post-processing:


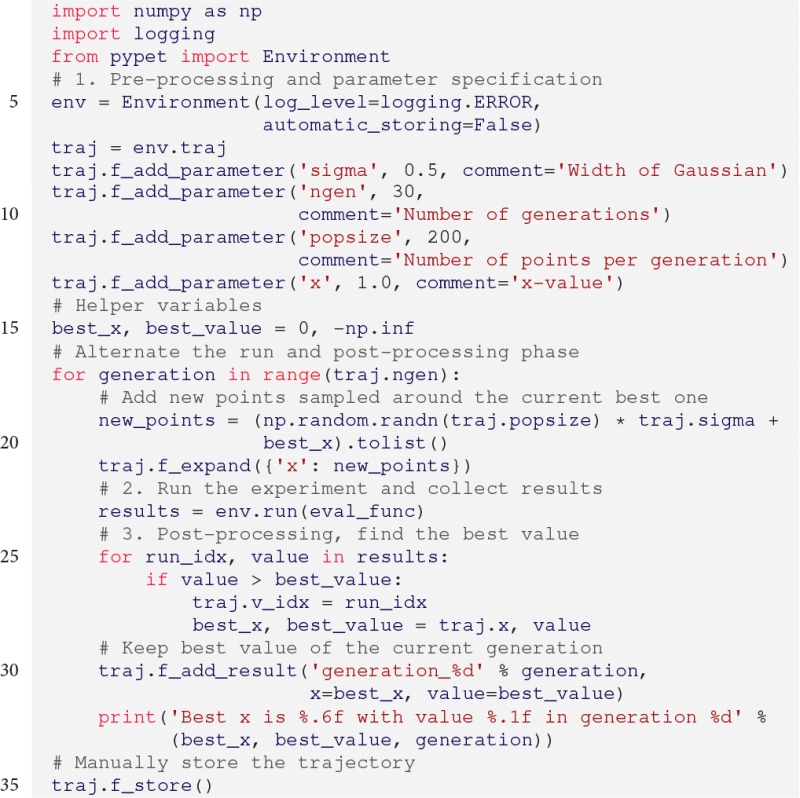


The keyword log_level = logging.ERROR (line 5) takes care that *pypet* only logs errors and keeps the output to the console to a minimum. In addition, via automatic_storing = False in line 6 *pypet* is told to not store data in regular intervals. Since we do not want to store any data during single runs, but process the results after each run phase, this statement saves some overhead.

Next, we iterate over 30 generations (traj.ngen) and sample 200 points (traj.popsize) in each generation from a Gaussian distribution (np.random.randn()) with a width of 0.5 (traj.sigma) centered at the current best point (lines 18–20). In every generation we expand the trajectory and add new points. In order to do so we can use the f_expand() function. It will either extend a Trajectory containing some already explored points or simply behave as the already known f_explore() function in case of an unexplored Trajectory at the initial loop iteration.

Subsequently, we obtain the results of the single runs. If our top-level simulation function returns data, the Environment will pass this data in form of a list of tuples back to the outer script (results in line 22). As mentioned before, each tuple has two entries: The first contains the index of the corresponding run and the second is the returned data. For example, in the first iteration the list may look like the following: [(0, 3342.267), (1, -9.42), (2, 4242.776), …].

Next, we perform the post-processing. We iterate through the obtained values and update the best point we found so far (lines 24–27). Additionally, we add the best point of each generation as a result to our Trajectory in line 30. At the end of the loop we print the current best point. Finally, because we turned off *pypet*'s automatic storing, we need to manually initiate the storing to disk (line 34).

If we run the above specified script, the best value of each generation will be printed to the screen:


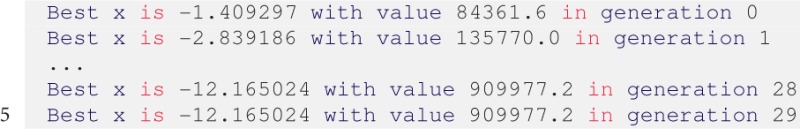


In general, the user does not have to wait until all single runs are finished to start post-processing. With multiprocessing *pypet* can already initiate post-processing and extend a Trajectory while the Environment still performs single runs. This feature of immediate post-processing is explained in the online documentation.

### 3.6. *pypet* and BRIAN2

We will demonstrate how to use *pypet* with the neuron simulator BRIAN2 (Stimberg et al., 2014). We will simulate a homogeneous population of neurons that are randomly coupled via inhibitory synapses. Each neuron obeys the following differential equation and spiking condition:

(3) $$\frac{dV_{i}}{dt}=\frac{1}{\tau }(I_{0}-V_{i})+\sum_{t_{j}}w\delta (t-t_{j}),$$

(4) $$\text{if }V_{i}\geq V_{T}:\text{ spike event }\&\;V_{i}\to 0,$$

with *V*_*i*_ denoting the dynamic membrane voltage of the *i*th neuron, τ is the membrane time constant, and *I*_0_ the static input. If the membrane voltage crosses the threshold of *V*_*T*_, it is reset to 0 and the event is counted as a spike. The neurons are randomly connected with a fixed probability. In case of a connection between neuron *j* and *i*, a pre-synaptic spike of neuron *j* at time *t*_*j*_ causes an instantaneous change of the membrane voltage of magnitude *w*. We will only consider inhibitory connections, i.e., *w* < 0.

First, we define a function to add all parameters of the model:


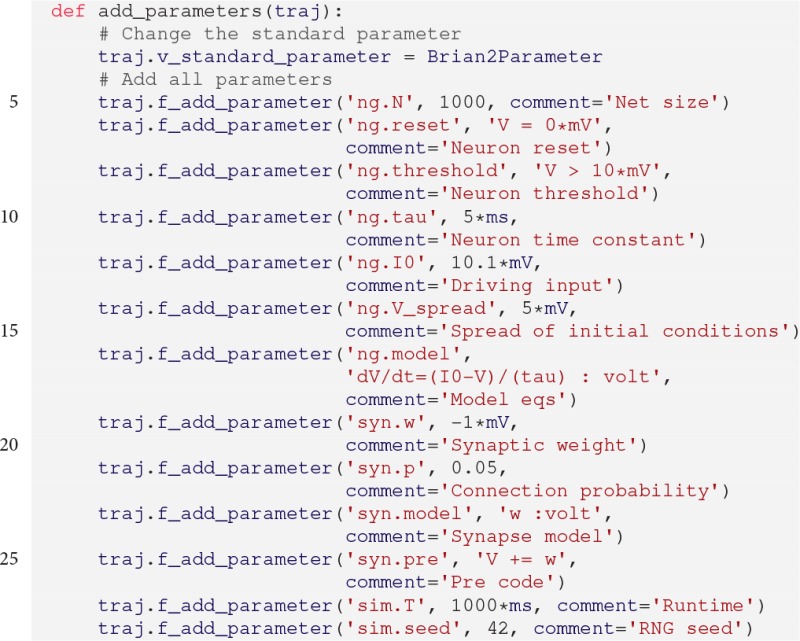


BRIAN2 supports quantities that have units like volt or ampere. Accordingly, we cannot use *pypet*'s default Parameter because it does not handle such data. However, there exists the specialized sub-class Brian2Parameter that supports BRIAN2 quantities. By setting traj.v_standard_parameter = Brian2Parameter in line 3, *pypet* will always use the Brian2Parameter instead of the Parameter.

Moreover, because the simulation is based on more than a few parameters, we structure our parameter space and sort the parameters into different sub-groups. For example, the synaptic weight 'w' is part of the 'syn' (short for synapse) group. Accordingly, the parameter addition traj.f_add_parameter('syn.w', … ) will automatically create the 'syn' group if it does not yet exist in the trajectory tree.

Next, the simulation function is given below. We create a BRIAN2 NeuronGroup with random membrane voltages as initial condition, add connections via Synapses, and record activity using a SpikeMonitor and voltage traces via a StateMonitor. Finally, after the network is run, the monitor data is handed over to the Trajectory:


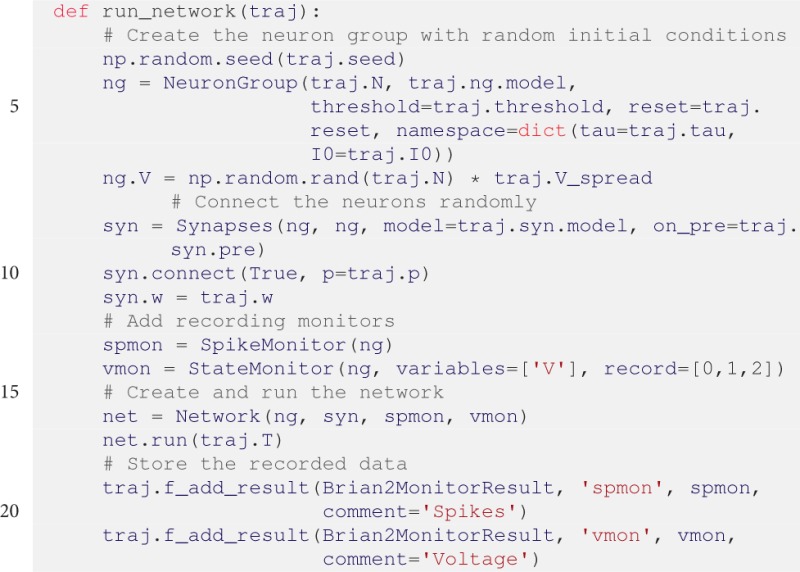


As before, BRIAN2 monitor data cannot be handled by the default Result. Accordingly, we use the Brian2MonitorResult that automatically extracts the recorded data of the monitors. In case of the SpikeMonitor the spike times are provided by an array spmon.t and the corresponding neuron indices by spmon.i. The extracted data is stored in the Trajectory under the same name. For example, the indices can be accessed via traj.results.runs.crun.spmon.i. Similarly, the data provided by the StateMonitor is the membrane voltage vmon.V and the measurement times vmon.t.

To run the simulation we still need some boilerplate code. Furthermore, we decide to explore different inhibitory synaptic connection strengths *w*:


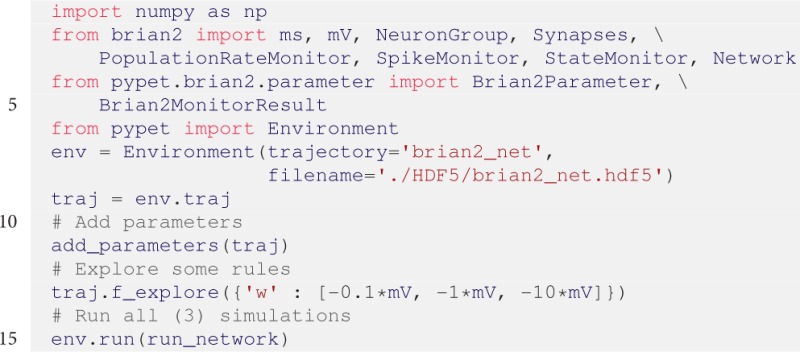


After the execution of the code above, we can plot the results in a new Python script. The script below plots the spiking activity as a raster plot and the voltage trace of one neuron for each run:


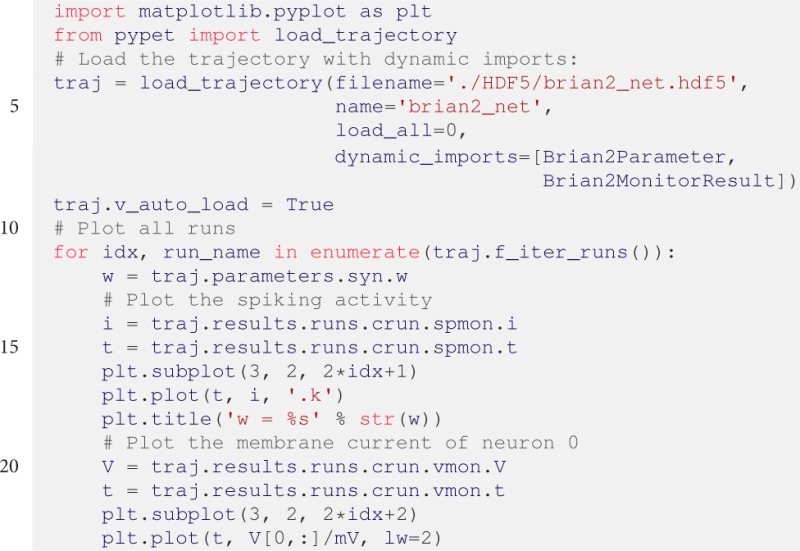


The corresponding plots are shown on the right hand side of Figure 3.

The keyword dynamic_imports=[Brian2Parameter, Brian2MonitorResult] in line 7 is needed because all parameters and results are handled by Brian2MonitorResult and Brian2MonitorResult containers. The Trajectory needs access to the container constructors Brian2Parameter and Brian2Parameter during runtime because the trajectory.py module has no direct access to BRIAN2 related elements to avoid a dependency on the BRIAN2 package. Accordingly, users can import *pypet* without the requirement of a BRIAN2 installation.

Similarly, if the user had written her own custom containers, for example named CustomResult or CustomParameter, these constructors should be passed to the Trajectory via dynamic_imports=[CustomResult, CustomParameter] as well. This allows *pypet* to appropriately load data for the customized containers from disk.

### 3.7. Integration with other software

**Figure 4:**
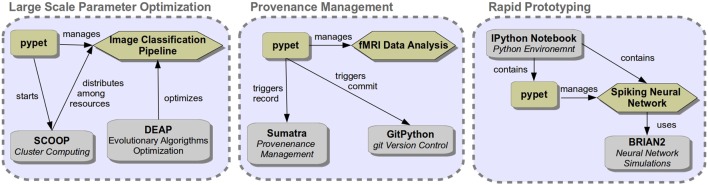
**Three use case examples combining ***pypet*** with other software**.

*pypet* can be combined with other packages depending on the research demands at hand. Figure 4 shows some exemplary use cases and the corresponding software setup.

A combination of *pypet*, SCOOP (Hold-Geoffroy et al., 2014), and the evolutionary algorithm toolkit DEAP (Fortin et al., 2012) could be used to optimize hyper parameters of a machine learning application, like image classification. Accordingly, *pypet* will use the SCOOP package to distribute runs among a server infrastructure for massively parallel exploration. In order to combine *pypet* with SCOOP one simply needs to create an environment in one's main Python script as follows:


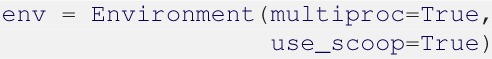


and start the script, here named mysimulation.py, with the - m scoop option:


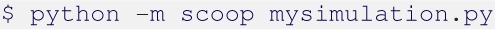


For details on how to choose SCOOP workers among multiple servers, how to use *pypet* with SCOOP on a computing cluster like a sun grid engine, and how to include DEAP into a *pypet* project, the reader is directed to the online documentation.

Another use case of *pypet* could be the analysis of experimental data, for instance fMRI time series data of brain scans. Analysis steps might be involved and rely on many parameters. In this case provenance management of a developing analysis pipeline can be important. Therefore, *pypet* could be combined with GitPython^17^ for source code version control and with Sumatra (Davison, 2012) to track versions of all applied software.

Accordingly, if the path to the main folder of the user's git repository is passed to the Environment via git_repository = '/path/to/project', *pypet* automatically triggers a git commit via GitPython if it finds changes in the code base. *pypet* remembers the commit's SHA identifier. Consequently, the user can always recall the exact version of a code base with which particular results were obtained. Instead of automatic commits, *pypet* can also be configured to raise an error in case of code changes via passing git_fail = True to the Environment.

Likewise, if the user's project is under the supervision of Sumatra, she can specify the path to the Sumatra project folder via passing sumatra_project = '/path/to/project' as keyword argument to the Environment constructor. Accordingly, *pypet* automatically submits a Sumatra record for provenance management. The Sumatra record will contain information about the computing platform, like the operating system, and the version numbers of all project's software dependencies.

*pypet* can also be used in small scripts for rapid prototyping. For instance, one may use *pypet* within an IPython notebook to develop a spiking neural network model based on the BRIAN2 simulator. IPython notebooks are especially suitable for fast prototyping. Plots of results can be displayed right next to the corresponding source code within the notebook. In addition, researchers can use the *pypet* Trajectory container interactively, for example by browsing the data tree using tab-completion.

## 4. Summary and discussion

We described *pypet*, a flexible Python tool to manage numerical experiments and simulations. *pypet* has a rich set of features and its main objectives are easy exploration of high dimensional parameter spaces and fostering ties between the parameters and simulation results.

*pypet* provides a novel container called Trajectory that governs all parameters and results. The data handled by a Trajectory is automatically stored to disk in the convenient HDF5 format. The tree structure of the Trajectory maps one-to-one to the data hierarchy in a HDF5 file.

In addition, *pypet*'s Environment forms a general framework for simulations. It schedules individual runs of the user's experiments, manages administrative tasks like logging, and can be used to parallelize simulations using multiple CPUs.

*pypet* integrates well with other libraries for advanced an extended usage. We demonstrated that *pypet* can be easily combined with git version control and the Sumatra library for comprehensive provenance management. We also sketched how to use *pypet* in a cluster or multi-server environment with SCOOP. Furthermore, in case the user wants to adaptively explore the parameter space, she can use the optimization toolbox DEAP, a Python framework for evolutionary algorithms.

In conclusion, by supporting data management via various features and by tightly linking numerical results and the underlying parameters, *pypet* enhances reproducible research in computational neuroscience and other disciplines exercising computer simulations in Python.

### 4.1. Limitations and future work

As with all software tools, *pypet* has its limitations. *pypet* adds some overhead to a user's simulation. On a conventional notebook *pypet*'s overhead adds roughly about 0.0001–0.1 s runtime to a single run. Of course, exact values depend on the hardware at hand, choices of parallelization, and how much data is stored. For simulations lasting seconds, minutes, or longer—which is more the rule than the exception in computational neuroscience—this pales into insignificance. Yet, for simulations with more than a million runs *pypet*'s overhead accumulates and can be a matter of days. Likewise, for this order of magnitude the overhead caused by run and parameter meta-data becomes a problem, too. Already loading explored parameters as well as run information data can take up to several seconds. This initial loading time makes analyses cumbersome. In this case the user is advised to split the runs across several trajectories and analyze the data therein independently.

**Figure 5:**
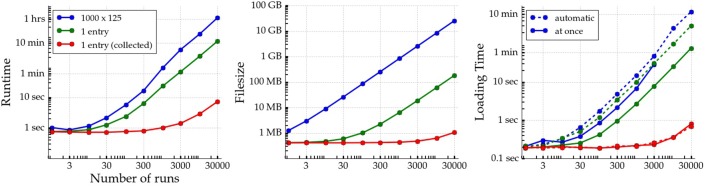
**Runtime, resulting HDF5 file size, and loading time of three simple ***pypet*** use cases are depicted as a function of the number of runs in an experiment**. All experiments involve only creating random numbers (via NumPy's random.rand() function) and storing the data to disk collectively at the end or individually in each run. In red a single run **returns** a random number that is stored together with all results after the execution of all single runs. In green a single run involves storing a single random floating point number (datasize 64 bit per number) in each run. In blue a large array of 1000 by 125 random numbers (datasize 1 megabyte per array) is written to disk in every run. On the right two different methods of *pypet*'s data loading are depicted for each experiment. The solid lines mark loading all data at once into memory. Note that in case of the 1000 by 125 arrays data could only be loaded at once for up to 3000 runs because of limited RAM. The dashed lines mark loading data via the v_auto_loading functionality iteratively. After loading data of a single run, it is immediately removed again to free RAM. Experiments were performed with a single core of 2.5 GHz on a conventional notebook (Lenovo ThinkPad T420) with 8 GB RAM, Samsung SSD 840 PRO, and Ubunutu 14.04 Linux operating system.

Furthermore, there exists overhead not only related to simulation meta-data and explored parameters, but also to results stored into an HDF5 file. The runtime, HDF5 file size, and loading time for three simple *pypet* use cases that involve creation and storage of random floating point numbers are depicted in Figure 5. *pypet* is well suited for experiments where individual runs already produce some considerable amount of data. In this case the overhead for scattering data across the HDF5 file is minuscule in comparison to the data itself. An example of such data is shown in blue in Figure 5 where an array of random numbers of 1 megabyte in size is stored in each run. In real experiments data could be arrays containing time series: Voltage traces of simulated neurons, for example. If the result of a single run is only a single number (green lines in Figure 5; see also the basic example in Section 3.3), however, it might be more useful to return each number. Subsequently, all returned numbers can be stored together into a single array after the runs are executed (red lines) to avoid the overhead induced by scattering many individual floats across the HDF5 file.

Furthermore, *pypet* relies on PyTables. PyTables does not support parallel access to HDF5 files. Even for massively parallel runs, data is only stored sequentially. Hence, if the storage of data makes up a large part of a single run, the data storage constitutes a bottleneck. However, *pypet* is modularized. The Trajectory and the containers are independent of the storage backend. Thus, besides the current HDF5StorageService, in the future *pypet* may be complemented with a service that allows parallel storage.

Likewise, HDF5 is an adequate format in case data is read often but only written once. Deleting or replacing existing data is possible but not well supported. The deletion of data does not reduce the file size. Accordingly, many deletions or data replacements may blow up the HDF5 file size considerably. However, for most of *pypet*'s intended use cases this does not constitute a major problem. *pypet* is designed for parameter exploration in numerical experiments. Accordingly, the results should be considered as experimental raw data with no need to change after the recording. Hence, data is only written once to an HDF5 file and not modified or overwritten afterwards. Still, in the future the inflexibility of the HDF5 format could be overcome by the implementation of a new backend, like a service supporting SQL or Mongo^18^ databases. Accordingly, the user can choose the backend that caters best to her needs.

## Author contributions

RM did the program design, implementation, and drafting the manuscript. KO did drafting and revising the manuscript and helped with the concept and design decisions.

## Funding

This work was funded by the Deutsche Forschungsgemeinschaft (GRK1589/1).

### Conflict of interest statement

The authors declare that the research was conducted in the absence of any commercial or financial relationships that could be construed as a potential conflict of interest.

## Footnotes

^1^https://www.hdfgroup.org/HDF5/

^2^https://www.hdfgroup.org/HDF5/

^3^http://www.pytables.org/

^4^In the Python universe the term *package* denotes a bundle of *modules*. Modules are specialized Python libraries that may contain related functions, objects, and general Python statements.

^5^In object oriented programming user interactions involve so called objects. These objects are special data structures that provide data in terms of object attributes. In addition, the object's methods grant functionality to modify the object and data therein.

^6^https://github.com/SmokinCaterpillar/pypet

^7^https://pypi.python.org/pypi/pypet

^8^https://travis-ci.org/SmokinCaterpillar/pypet

^9^https://ci.appveyor.com/project/SmokinCaterpillar/pypet

^10^https://coveralls.io/github/SmokinCaterpillar/pypet

^11^https://www.mongodb.com/

^12^http://www.pytables.org/

^13^http://www.hdfgroup.org/products/java/hdfview/

^14^https://pypet.readthedocs.org/en/latest/

^15^From Python version 3.4 on, *pip* is part of the Python distribution and is no longer needed to be installed manually.

^16^https://pypi.python.org/pypi/pypet

^17^https://gitpython.readthedocs.org

^18^https://www.mongodb.com/
